# Polymorphism of the *Flap Endonuclease 1* Gene in Keratoconus and Fuchs Endothelial Corneal Dystrophy

**DOI:** 10.3390/ijms150814786

**Published:** 2014-08-22

**Authors:** Katarzyna A. Wojcik, Ewelina Synowiec, Piotr Polakowski, Sylwester Głowacki, Justyna Izdebska, Sophie Lloyd, Dieter Galea, Janusz Blasiak, Jerzy Szaflik, Jacek P. Szaflik

**Affiliations:** 1 Department of Molecular Genetics, University of Lodz, Pomorska 141/143, Lodz 90-236, Poland; E-Mails: kwojcik@biol.uni.lodz.pl (K.A.W.); ewelinas@biol.uni.lodz.pl (E.S.); sglowa@biol.uni.lodz.pl (S.G.); jblasiak@biol.uni.lodz.pl (J.B.); 2 Department of Ophthalmology, Medical University of Warsaw, SPKSO Ophthalmic Hospital, Sierakowskiego 13, Warsaw 03-709, Poland; E-Mails: polakowp@gmail.com (P.P); justyna_izdebska@yahoo.es (J.I.); szaflik@szaflik.pl (J.S.); 3 School of Biosciences, Cardiff University, Museum Avenue, Cardiff, CF10 3AX, United Kingdom, on IESTE training at Department of Molecular Genetics, University of Lodz, Pomorska 141/143, Lodz 90-236, Poland; E-Mail: lloydsophieanne@gmail.com; 4 Department of Biology, University of Malta, Msida MSD2080, Malta, on IESTE training at Department of Molecular Genetics, University of Lodz, Pomorska 141/143, Lodz 90-236, Poland; E-Mail: dietergalea@gmail.com

**Keywords:** keratoconus, Fuchs endothelial corneal dystrophy, flap endonuclease 1, DNA repair

## Abstract

Oxidative stress is implicated in the pathogenesis of many diseases, including serious ocular diseases, keratoconus (KC) and Fuchs endothelial corneal dystrophy (FECD). Flap endonuclease 1 (FEN1) plays an important role in the repair of oxidative DNA damage in the base excision repair pathway. We determined the association between two single nucleotide polymorphisms (SNPs), c.–441G>A (rs174538) and g.61564299G>T (rs4246215), in the *FEN1* gene and the occurrence of KC and FECD. This study involved 279 patients with KC, 225 patients with FECD and 322 control individuals. Polymerase chain reaction (PCR) and length polymorphism restriction fragment analysis (RFLP) were applied. The T/T genotype of the g.61564299G>T polymorphism was associated with an increased occurrence of KC and FECD. There was no association between the c.–441G>A polymorphism and either disease. However, the GG haplotype of both polymorphisms was observed more frequently and the GT haplotype less frequently in the KC group than the control. The AG haplotype was associated with increased FECD occurrence. Our findings suggest that the g.61564299G>T and c.–441G>A polymorphisms in the *FEN1* gene may modulate the risk of keratoconus and Fuchs endothelial corneal dystrophy.

## 1. Introduction

As an outer layer of the eye, the cornea is especially susceptible to environmental factors. Exposure to blue light and ultraviolet (UV) radiation, as well as mechanical trauma, resulting from chronic eye rubbing and contact lens wearing, may produce reactive oxygen species (ROS) and induce oxidative stress in it [1]. Oxidative stress is implicated in the pathogenesis of several ocular diseases, including keratoconus (KC) and Fuchs endothelial corneal dystrophy (FECD) [2,3].

Keratoconus is a degenerative disorder of the eye, characterized by a progressive thinning of the corneal stroma and loss of normal corneal architecture, which assumes a conical shape [4]. Other signs include breaks in Bowman’s membrane, deposition of iron in the basal layers of the corneal epithelium and the formation of fine parallel lines in the posterior stroma [5]. Keratoconus typically occurs at puberty and progresses until the third or fourth decade of life. Clinical signs differ depending on the stage of the disease [4,5]. In the early cases, KC is asymptomatic; however, further development leads to a distortion of vision and image blurring [6,7]. A prevalence of KC ranges from 1.3 to 25 per 100,000 in the general population [8,9]. Despite numerous investigations, the aetiology of KC is not completely understood. It is suspected that multiple genes together with environmental factors contribute to its pathogenesis [10]. Although KC is usually a sporadic disease, in some cases, autosomal dominant inheritance was noticed. Prevalence of KC in first degree relatives is 15–67-times higher than in the general population, indicating the importance of the genetic trait in its aetiology [9]. A genetic basis for KC is also supported by high concordance in monozygotic twins [11,12]. Several genes were proposed as candidate genes for KC, including different types of collagen (COL4A3 and COL4A4), proteinase inhibitors (*TIMP3*), as well as antioxidant genes (*SOD1*) and genes belonging to the homeobox family (*VSX1*) [13,14,15]. Moreover, linkage studies carried out in families affected by KC detected chromosomal loci associated with this disease, including: 3p14-q13; 5q14-q21; 15q22-q24; 1p36; 8q13-q21, 2p24; 16q22-q23; 13q32; and 20q12 [16,17,18,19,20,21,22,23]. However, few causative mutations were identified in these regions.

FECD is a disorder of corneal endothelium. This disease is characterized by a decrease in endothelial cell density, deposition of Descemet’s membrane, termed guttae, and changes in endothelial cell morphology [24,25]. The loss of corneal endothelial cells causes a disturbance of the fluid balance in this tissue, which results in decreased visual acuity. FECD brings on blurred vision, which progresses as the disease progresses, and in the late stages, it can cause blindness [26]. The prevalence of FECD in individuals over 40 is approximately 4% [27]. Currently, corneal transplantation is the only treatment modality to restore lost vision [28]. FECD is a common cause of corneal transplantation in the United States for patients over 60 [28,29]. The pathogenesis of this disease is complex and results from the interaction between genetic and environment factors [3,28]. The disorder is classified into two forms: early and late onset [30]. The early-onset form is rare and begins in early middle age. This form is linked with mutations in *COL8A2*, which encodes the collagen helix domain of the α2 chain of type VIII collagen, a main component of Descemet’s membrane [31,32]. The late-onset FECD is more common and typically appears in the fifth decade of life [26]. To date, three genes, *ZEB1* (*TCF8*), encoding the zinc finger E-box-binding homeobox 1 transcription factor, *SLC4A11*, coding for a sodium-borate cotransporter, and *LOXHD1*, encoding lipoxygenase homology domain 1 were found to be involved in the development of more common late-onset FECD [33,34,35]. In addition, several chromosomal loci were associated with late-onset FECD, including FCD1 at 13pTel-13q12.13, FCD2 at 18q21.2-q21.32, FCD3 described at 5q33.1-q35.2 and FCD4 at 9p24.1e22.1 [34,36,37,38,39]. A linkage study also identified chromosomes 1, 7, 15, 17 and X as potentially involved in the development of FECD [38].

Genetic factors in KC and FECD were intensively investigated in recent years [9,28,40,41]. In this work, we checked whether polymorphisms of the *flap endonuclease 1* (*FEN1*) gene may be associated with KC or FECD occurrence. FEN1 plays an essential role in the repair DNA damage during long-patch base-excision repair (BER), so changes in its gene may lead to the increased susceptibility of DNA to oxidative injury. The results of several studies indicate a role for oxidative stress in the development of KC and FECD. We hypothesize that the variability in the *FEN1* gene may change the susceptibility to oxidative stress and contribute to the development of KC and FECD. To verify this hypothesis, we checked the association between the c.–441G>A (rs174538) and g.61564299G>T (rs4246215) polymorphisms and KC/FECD occurrence, as well as the modulation of this association by some demographic and risk factors for KC/FECD.

## 2. Results

### 2.1. Characteristics of Study Subjects

Demographic variables and potential risk factors for KC and FECD of the study patients and controls are shown in Table 1. There were significantly more subjects with a positive family history for KC and FECD (first degree relatives) among the patients in comparison to controls (11% and 16%* vs.* 3%, *p* < 0.001). We showed a significant differences between the distribution of family history for KC/FECD (positive *vs.* negative family history) and co-occurrence of visual impairment (yes* vs.* no) among KC/FECD patients and controls. We also demonstrated a significant differences between the distribution of allergies (yes* vs.* never) and heart or vascular diseases (yes* vs.* never) and co-occurrence of visual impairment (yes *vs.* no) among KC patients and controls. These parameters were further adjusted in a multivariate logistic regression model for possible confounding factors of the main effect of the single nucleotide polymorphisms (SNPs).

**Table 1:** Characteristics of keratoconus (KC) and Fuchs endothelial corneal dystrophy (FECD) patients and controls enrolled in this study.

Feature	Controls (*n* = 322)		KC (*n* = 279)		*p*	FECD (*n* = 225)		*p*
	Number	Frequency	Number	Frequency		Number	Frequency	
**Sex**								
Females	205	0.64	84	0.30	**<0.001**	172	0.76	**0.002**
Males	117	0.36	195	0.70		53	0.24	
**Age**								
Mean ± SD	63.78 ± 18.82		36.33 ± 12.08		<0.001 *	70.52 ± 9.81		<0.001 *
Range	19–100		14–68			37–91		
**Smoking**								
Yes (current/former)	107	0.33	88	0.32	0.724	78	0.35	0.797
Never	215	0.67	191	0.68		147	0.65	
**KC/FECD in family**								
Yes	9	0.03	31	0.11	**0.001**	36	0.16	**<0.001**
No	313	0.97	248	0.89		189	0.84	
**BMI**								
≤25	130	0.41	127	0.45	0.469	93	0.41	0.939
25–30	114	0.35	91	0.33		77	0.34	
≥30	78	0.24	61	0.22		55	0.25	
**Visual impairment**								
Yes	103	0.32	195	0.70	**<0.001**	123	0.55	**<0.001**
No	219	0.68	84	0.30		102	0.44	
**Allergies**								
Yes	40	0.12	77	0.28	**<0.001**	40	0.18	0.105
No	282	0.88	202	0.72		185	0.82	
**Heart and vascular diseases**								
Yes	177	0.55	58	0.21	**<0.001**	130	0.58	0.573
No	145	0.45	221	0.79		95	0.42	

*p*-values for two-sided χ^2^ test, except: * *p*-values for *t*-test; and *p* < 0.05 are in bold.

### 2.2. Relationship between Age, Sex, Tobacco Smoking, Co-Occurrence of Visual Disturbances, BMI, Heart and Vascular Diseases, Allergies and KC/FECD in Family and the Risk of KC/FECD Independent of Genotype

We analysed the relationships between age, sex, tobacco smoking, co-occurrence of visual disturbances, body mass index (BMI), heart and vascular diseases, allergies and KC/FECD in family and the risk of KC/FECD independently of genotype. We compared KC and FECD patients with controls according to these parameters (Table 2 and Table 3). Male sex, KC in family, co-occurrence of visual disturbances and allergies significantly increased the occurrence of KC, whereas age and co-occurrence of heart and vascular diseases decreased this occurrence. We also found that female sex, age, FECD in family and co-existence of visual disturbances significantly increased the occurrence of FECD.

**Table 2:** Risk of KC associated with age, sex, tobacco smoking, co-occurrence of visual disturbances, body mass index (BMI), heart and vascular diseases, allergies and family history of keratoconus (KC).

Characteristics	Controls		KC		OR (95% CI)	*p*
	Number	Frequency	Number	Frequency		
**Sex**						
Females	205	0.64	84	0.30	**0.25 (0.17–0.35)**	**<0.001**
Males	117	0.36	195	0.70	**4.07 (2.89–5.72)**	**<0.001**
**Age**	63.78 ± 18.82		36.33 ± 12.08		**0.91 (0.90–0.93)**	**<0.001**
**Smoking**						
Yes (current/former)	107	0.33	88	0.32	0.94 (0.66–1.32)	0.710
Never	215	0.67	191	0.68	1.07 (0.76–1.50)	0.710
**KC in family**						
Yes	6	0.02	31	0.11	**6.52 (2.68–15.89)**	**<0.001**
No	316	0.98	248	0.89	**0.15 (0.06–0.37)**	**<0.001**
**BMI**						
≤25	130	0.41	127	0.45	1.23 (0.89–1.70)	0.219
25–30	114	0.35	91	0.33	0.88 (0.63–1.24)	0.470
≥30	78	0.24	61	0.22	0.88 (0.60–1.30)	0.529
**Visual impairment**						
Yes	103	0.32	195	0.70	**4.87 (3.43–6.91)**	**<0.001**
No	219	0.68	84	0.30	**0.20 (0.14–0.29)**	**<0.001**
**Allergies**						
Yes	40	0.12	77	0.28	**2.62 (1.72–3.99)**	**<0.001**
No	282	0.88	202	0.72	**0.38 (0.25–0.58)**	**<0.001**
**Heart and vascular diseases**						
Yes	177	0.55	58	0.21	**0.22 (0.15–0.31)**	**<0.001**
No	145	0.45	221	0.79	**4.63 (3.22–6.66)**	**<0.001**

OR, odds ratio; 95% CI, 95% confidence interval; *p*-values < 0.05 along with corresponding ORs are in bold.

**Table 3:** Risk of Fuchs endothelial corneal dystrophy (FECD) associated with age, sex, tobacco smoking, co-occurrence of visual disturbances, body mass index (BMI), heart and vascular diseases, allergies and family history of FECD.

Characteristics	Controls		FECD		OR (95% CI)	*p*
	Number	Frequency	Number	Frequency		
**Sex **						
Females	205	0.64	172	0.76	**1.85 (1.26–2.71)**	**0.002**
Males	117	0.36	53	0.24	**0.54 (0.37–0.79)**	**0.002**
**Age**	63.78 ± 18.82		70.52 ± 9.81		**1.03 (1.01–1.04)**	**<0.001**
**Smoking**						
Yes (current/former)	107	0.33	78	0.35	1.08 (0.75–1.55)	0.682
Never	215	0.67	147	0.65	0.93 (0.65–1.33)	0.682
**FECD in family **						
Yes	3	0.01	36	0.16	**20.54 (6.24–67.65)**	**<0.001**
No	319	0.99	189	0.84	**0.04 (0.01–0.16)**	**<0.001**
**BMI**						
≤25	130	0.41	93	0.41	1.04 (0.73–1.47)	0.827
25–30	114	0.35	77	0.34	0.94 (0.65–1.34)	0.724
≥30	78	0.24	55	0.25	1.03 (0.69–1.53)	0.886
**Visual impairment**						
Yes	103	0.32	123	0.55	**3.16 (2.21–4.53)**	**<0.001**
No	219	0.68	102	0.44	**0.32 (0.22–0.45)**	**<0.001**
**Allergies**						
Yes	40	0.12	40	0.18	1.56 (0.97–2.51)	0.068
No	282	0.88	185	0.82	0.64 (0.40–1.03)	0.068
**Heart and vascular diseases**						
Yes	177	0.55	130	0.58	1.14 (0.81–1.62)	0.441
No	145	0.45	95	0.42	0.87 (0.62–1.23)	0.441

OR, odds ratio; 95% CI, 95% confidence interval; *p*-values < 0.05 along with corresponding ORs are in bold.

### 2.3. The c.–441G>A and the g.61564299G>T Polymorphisms of the FEN1 Gene and KC/FECD Occurrence

The genotype and allele distributions of the c.–441G>A and the g.61564299G>T polymorphisms of the *FEN1* gene in KC patients and controls are presented in Table 4. We observed a significant (*p* < 0.05) difference in the frequency distributions of genotypes of the g.61564299G>T polymorphism between the cases and controls. The presence of the T/T genotype was associated with an increased occurrence of KC. We did not find any association between genotypes/alleles of the c.–441G*>*A polymorphism and KC occurrence.

**Table 4:** Distribution of genotypes and alleles of the c.–441G>A (rs174538) and the g.61564299G>T (rs4246215) polymorphisms of the *FEN1* gene and the odds ratio (OR) with the 95% confidence interval (95% CI) in patients with keratoconus (KC) and controls.

Polymorphism Genotype/Allele	Controls (*n* = 322)		KC (*n* = 279)		Crude OR (95% CI)	*p*	Adjusted OR ^a^ (95% CI)	*p*
	Number	Frequency	Number	Frequency				
c.-441G>A								
A/A	17	0.05	17	0.06	1.16 (0.58–2.33)	0.667	1.28 (0.38–4.25)	0.689
A/G	178	0.55	161	0.58	1.10 (0.80–1.52)	0.550	0.64 (0.40–1.05)	0.079
G/G	127	0.39	101	0.36	0.87 (0.63–1.21)	0.414	1.52 (0.92–2.51)	0.104
χ^2^ = 0.745; *p* = 0.6891								
A	212	0.33	195	0.35	1.13 (0.85–1.49)	0.390	0.75 (0.49–1.16)	0.200
G	432	0.67	363	0.65	0.88 (0.67–1.17)	0.390	1.33 (0.86–2.06)	0.200
g.61564299G>T								
G/G	149	0.46	106	0.38	**0.71 (0.51–0.99)**	**0.042**	0.96 (0.59–1.55)	0.860
G/T	157	0.49	141	0.51	1.07 (0.78–1.48)	0.663	0.74 (0.46–1.20)	0.219
T/T	16	0.05	32	0.11	**2.48 (1.33–4.62)**	**0.004**	**5.15 (1.69–15.67)**	**0.004**
χ^2^ = 10.420; *p* = 0.0055								
G	455	0.71	353	0.63	**0.68 (0.52–0.88)**	**0.004**	0.76 (0.51–1.14)	0.185
T	189	0.29	205	0.37	**1.47 (1.13–1.91)**	**0.004**	1.31 (0.88–1.96)	0.185

*p* < 0.05 along with corresponding ORs are in bold; OR ^a^ adjusted for sex, age, co-occurrence of visual impairment, allergies, heart and vascular diseases and family history for KC.

Table 5 presents the genotype and allele distributions of the c.–441G>A and the g.61564299G>T polymorphisms of the *FEN1* gene in FECD patients and controls. We detected a significant (*p* < 0.05) difference in the frequency distributions of genotypes of the c.–441G>A polymorphism between the cases and controls. We showed an association between the presence of the T/T genotype of the g.61564299G>T polymorphism and an increase of the occurrence of FECD. We did not detect any correlation between genotypes/alleles of the c.–441G>A polymorphism and FECD occurrence.

**Table 5:** Distribution of genotypes and alleles of the c.–441G>A (rs174538) and the g.61564299G>T (rs4246215) polymorphisms of the *FEN1* gene and odds ratio (OR) with 95% confidence interval (95% CI) in patients with Fuchs endothelial corneal dystrophy (FECD) and controls.

Polymorphism Genotype/Allele	Controls (*n* = 322)		FECD (*n* = 225)		Crude OR (95% CI)	*p*	Adjusted OR ^a^ (95% CI)	*p*
	Number	Frequency	Number	Frequency				
c.-441G>A								
A/A	17	0.05	12	0.05	1.01 (0.47–2.16)	0.978	1.20 (0.47–3.07)	0.706
A/G	178	0.55	101	0.45	**0.66 (0.47–0.93)**	**0.017**	0.73 (0.48–1.10)	0.134
G/G	127	0.39	112	0.50	**1.52 (1.08–2.15)**	**0.017**	1.33 (0.88–2.00)	0.179
χ^2^ = 6.043; *p* = 0.0487								
A	212	0.33	125	0.28	**0.74 (0.55–0.99)**	**0.044**	0.83 (0.58–1.19)	0.312
G	432	0.67	325	0.72	**1.35 (1.01–1.82)**	**0.044**	1.20 (0.84–1.71)	0.312
g.61564299G>T								
G/G	149	0.46	106	0.47	1.03 (0.73–1.45)	0.847	0.96 (0.64–1.45)	0.859
G/T	157	0.49	98	0.44	0.81 (0.57–1.14)	0.230	0.84 (0.55–1.26)	0.349
T/T	16	0.05	21	0.09	**1.97 (1.01–3.86)**	**0.049**	**2.25 (1.01–5.00)**	**0.047**
χ^2^ = 4.519; *p* = 0.1040								
G	455	0.71	310	0.69	0.91 (0.69–1.20)	0.508	0.85 (0.61–1.18)	0.329
T	189	0.29	140	0.31	1.10 (0.83–1.45)	0.508	1.18 (0.85–1.64)	0.329
	17	0.05						

*p*-values < 0.05 along with corresponding ORs are in bold; OR ^a^ adjusted for the co-occurrence of visual impairment, sex, age and family history for FECD.

The observed genotypes frequencies of the g.61564299G>T polymorphism did not deviate statistically significantly from these expected from the Hardy–Weinberg equilibrium, among KC and FECD patients (*p* > 0.05, data not shown).

### 2.4. Haplotypes and KC/FECD Occurrence

The association between KC/FECD and haplotypes of the c.–441G>A and the g.61564299G>T polymorphisms of the *FEN1* gene was also assessed (Table 6). The presence of the GG haplotype was associated with increased and the GT haplotype with decreased occurrence of KC. We also detected an association between the AG haplotype and an increased occurrence of FECD.

**Table 6:** Distribution of haplotypes of c.–441G>A (rs174538) and the g.61564299G>T (rs4246215) polymorphisms of the *FEN1* gene and the odds ratio (OR) with the 95% confidence interval (95% CI) in patients with KC and FECD and controls.

Haplotype	Controls (*n* = 322)		KC (*n* = 279)		OR (95% CI)	*p*	FECD (*n* = 225)		OR (95% CI)	*p*
	Number	Frequency	Number	Frequency			Number	Frequency		
AG	221	0.17	178	0.16	1.09 (0.88–1.35)	0.427	110	0.12	**1.49 (1.16–1.90)**	**0.002**
AT	203	0.16	212	0.19	0.79 (0.65–0.99)	0.036	140	0.16	1.02 (0.80–1.28)	0.897
GG	689	0.53	528	0.47	**1.28 (1.10–1.50)**	**0.002**	510	0.57	0.88 (0.74–1.04)	0.142
GT	175	0.14	198	0.18	**0.73 (0.58–0.91)**	**0.051**	140	0.16	0.85 (0.67–1.09)	0.197

*p* < 0.05 along with corresponding ORs are in bold.

## 3. Discussion

KC and FECD are eye diseases with a complex aetiology. The pathogenesis of these diseases is still poorly understood, although several studies try to clarify mechanisms of their development and progression. To date, multiple genes were identified as possibly implicated in KC and FECD [9,28,40,41,42]. In addition, various environmental factors, including chronic eye rubbing, wearing hard contact lenses and atopy of the eye, seem to have a role in the pathogenesis of these diseases [3,26,43].

The analysis of the relationship between some clinical, environmental and life style parameters and the occurrence of KC and FECD independent from genotypes in our study indicated a significant influence of a positive family history of KC and FECD. A strong correlation between KC/FECD family history and KC/FECD occurrence was found in several other studies [9,44]. A significant association between visual impairment, allergic, heart and vascular disease and KC was also detected. In the present work, we demonstrated a significant correlation between visual impairment and FECD occurrence. These results confirm results obtained in other laboratories [43,45,46,47].

A growing body of evidence indicates that oxidative stress plays an important role in the pathogenesis of KC and FECD [3,48,49,50,51]. This hypothesis is supported by an excess of ROS and disturbance in the level of transcripts and/or activities of antioxidant enzymes in KC and FECD corneas compared to controls [3,48,49,51,52]. In addition, increased levels of oxidant-induced DNA damage in KC and FECD corneas was found, especially in mitochondrial DNA (mtDNA) [2,3,53].

An increased levels of oxidative DNA damage in KC and FECD corneas may be associated with functional changes in DNA repair genes that result in a decrease in the DNA repair capacity. Human cells have multiple DNA repair pathways, including nucleotide excision repair (NER), base-excision repair (BER), mismatch repair (MMR), homologous recombination repair (HRR) and non-homologous end joining (NHEJ).

FEN1 is an important nuclease, which is involved in the repair of non-bulky DNA lesions, including multiple oxidative DNA damage. FEN1 removes bifurcated DNA structures with displaced 5'-single-stranded DNA flaps during long-patch BER [54]. Moreover, this enzyme has 5' to 3' exonuclease and gap-dependent endonuclease activities, owing to its being implicated in the maturation of Okazaki fragments and the rescue of stalled replication forks [55]. FEN1 is also involved in other major DNA metabolic pathways, including resolution of tri-nucleotide repeat sequence-derived secondary structures, maintenance of telomere stability and apoptotic fragmentation of DNA [56]. FEN1 was also detected in the mitochondria, where it participates in mitochondrial DNA (mtDNA) replication and repair. As FEN1 has an important role in numerous DNA metabolic pathways, this enzyme is essential to maintain genome integrity. The functional deficiency of FEN1 may lead to severe genomic instability and an increased mutation rate, contributing to susceptibility to a number of genetic diseases [57]. It was found that the c.–441G>A SNP located in the *FEN1* promoter causes changes in promoter activity, whereas the g.61564299G>T SNP is associated with different levels of FEN1 RNA expression [58]. The c.–441G>A and the g.61564299G>T polymorphisms of *FEN1* are significantly associated with reduced FEN1 expression and increased DNA damage. It was found that these SNPs increase the risk of developing lung cancer in Chinese populations.

To our knowledge, this is the first study investigating the association of the c.–441G>A and the g.61564299G>T polymorphisms of the *FEN1* gene on the occurrence of KC and FECD. We found a significant association between the g.61564299G>T polymorphism of the *FEN1* gene and KC risk. The occurrence of KC was significantly increased in patients carrying the T/T genotype, suggesting that the g.61564299G>T polymorphism may play a role in KC. However, we observed no association between the c.–441G>A polymorphism of the *FEN1* gene polymorphism and the risk of KC. The T/T genotype of the g.61564299G>T polymorphism was also positively correlated with increased occurrence of FECD. We did not find any association between the c.–441G>A polymorphism of the *FEN1* gene and FECD.

## 4. Experimental Section 

### 4.1. Study Population

Our study was carried out on 279 patients with KC, 255 patients with FECD and 322 individuals with FECD/KC exclusion (controls). They were enrolled in the Department of Ophthalmology, Medical University of Warsaw (Warsaw, Poland). Medical history was obtained from all patients, and none of them had any genetic disease.

The diagnosis of KC was based on clinical signs and pachymetric and topographical parameters on Orbscan and Topographic Modeling System (TMS) corneal topography examinations [4,59,60]. The map patterns were carefully interpreted manually in all cases. All subjects underwent ophthalmic examination, including intraocular pressure, slit lamp examination, best-corrected visual acuity, fundus examination, Orbscan corneal topographical and pachymetrical maps (Orbscan IIz, Bausch & Lomb, Rochester, NY, USA) and corneal topography (TMS4, Tomey, Nagoya, Japan).

The diagnosis of FECD was based on clinical signs on the slit lamp examination, including the occurrence of endothelial guttae and corneal oedema. Diagnoses were confirmed by the presence of specific lesions, polymegathism and pleomorphism of the endothelial cells in* in vivo* confocal microscopy (IVCM) examination [61,62]. Patients underwent ophthalmic examination, including intraocular pressure, best-corrected visual acuity, fundus examination, slit lamp examination, IVCM and anterior segment optical coherence tomography (AS-OCT), including pachymetry maps. The AS-OCT was performed by Swept Source Anterior Segment Casia OCT (Tomey, Nagoya, Japan). The IVCM was performed by a white light scanning slit confocal microscopy system (ConfoScan 3 or ConfoScan 4, Nidek Techologies, Padova, Italy).

The control subjects showed no clinical evidence of FECD/KC and had healthy corneal endothelium on IVCM and normal corneal pachymetry and topography.

Each individual donated five millilitres of venous blood to EDTA-containing tubes, which were stored at −20 °C.

This study was performed according to the tenets of the Bioethics Committee of the Medical University of Warsaw. All patients gave written informed consent and were interviewed using a structural questionnaire to obtain information on demographic data and potential risk factors for KC and FECD. The data collected included age, BMI, co-occurrence of visual impairment (hyperopia, astigmatism, myopia), heart or vascular diseases, allergy, family history among first degree relatives for KC or FECD and lifestyle habits, including smoking. Smoking was categorized as never smokers and ever smokers. The characteristics of all subjects are presented in Table 1.

### 4.2. Selection of SNPs and Primer Design

The c.–441G>A and g.61564299G>T polymorphisms of the *FEN1* gene were chosen from the public domain of the National Centre for Biotechnology Information at http://www.ncbi.nlm.nih.gov/snp. These SNPs possess a minor allele frequency (MAF) 0.321 and 0.628 in the Caucasian population, respectively (submitter population ID: HapMap-CEU for both http://www.ncbi.nlm.nih.gov/snp). The primers were designed using the published nucleotide sequence in the ENSEMBL database (gene ID ENSG00000168496) and Primer3 software (http://frodo.wi.mit.edu/).

### 4.3. Genotyping

Total genomic DNA was isolated from peripheral blood leukocytes using the commercially available AxyPrep™ Blood Genomic DNA Miniprep kit (Axygen Biosciences, Union City, CA, USA), according to the manufacturer’s protocol. The purity and concentration of DNA were determined by taking the optical density (OD) of the samples at 260 and 280 nm, and the DNA was stored in TE buffer (5 mM Tris-HCl, 0.1 mM EDTA, pH 8.5), at −20 °C until use.

The polymerase chain reaction-restriction fragment length polymorphism (PCR-RFLP) method was used to genotype these polymorphisms of the *FEN1* gene. A PCR assay was carried out in a final reaction volume of 10 μL. The reaction mixture contained 25 ng of genomic DNA, 1× KAPA Taq Ready Mix containing KapaTaq DNA polymerase (0.025 U/μL), reaction buffer with MgCl_2_, 0.2 mM each dNTP (Kapa Biosystems, Woburn, MA, USA) and 0.25 μM each primer (Sigma-Aldrich, St. Louis, MO, USA).

**Figure 1:**
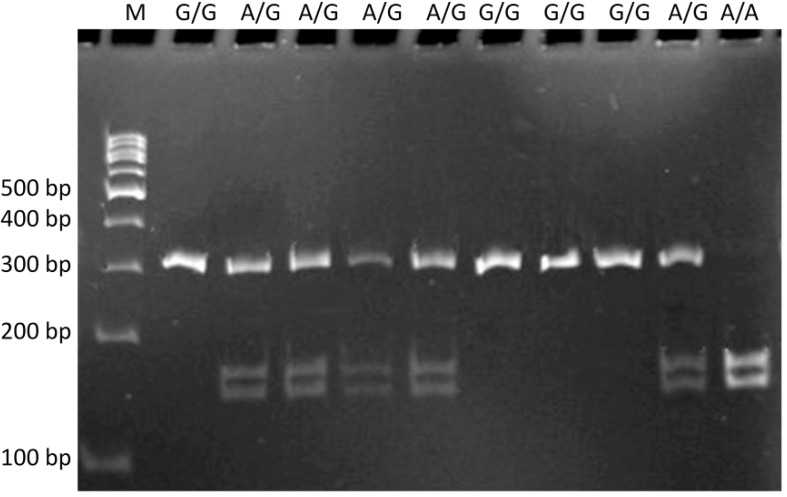
Restriction fragment length polymorphism analysis of the *FEN1* c.–441G>A (rs174538) polymorphism. Genotypes are indicated in the upper part of the picture. Lane M is a GeneRuler™ 100-bp marker ladder.

The following sequencing primers were used to detect the c.–441G>A SNP: the sense 5'-GGAGGTTCCAGGAGCGTCTA-3' and antisense 5'-TTCTCCACCGCTTGTCCC-3'. The PCR profile comprised a 5-min initial denaturation at 95 °C, 34 cycles of denaturation at 95 °C for 30 s, at 65 °C annealing for 30 s, extension for 60 s at 72 °C, and the final extension for 5 min at 72 °C. The amplified DNA fragments of 321 bp in length, containing the polymorphic site, were digested with 1.5 U of *Sal*I restriction endonuclease (New England Biolabs, Ipswich, UK) in a final volume of 15 μL for 16 h at 37 °C. Individual genotypes were assigned according to the size of the products: the G/G genotype corresponds to 321 bp; the A/A genotype 169 and 152 bp and the A/G genotype 321, 169 and 152 bp (Figure 1).

**Figure 2:**
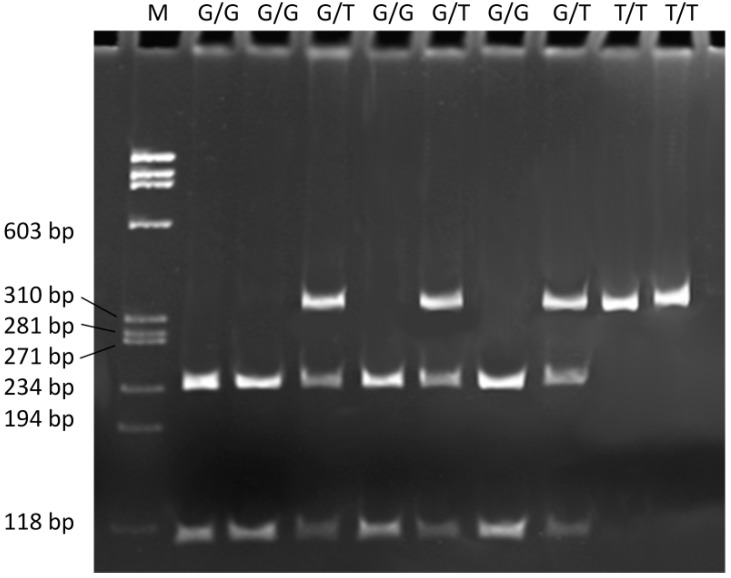
Restriction fragment length polymorphism analyses of the g.61564299G>T (rs4246215) polymorphism of *FEN1*. Genotypes are indicated in the upper part of the picture. Lane **M** is a ΦX174 DNA/BsuRI Marker ladder.

The g.61564299G>T polymorphism was genotyped in a fragment of the *FEN1* gene with the following primer sequences: forward 5'-TATGTCAGGCTCAAACCAC-3' and reverse 5'-CAGCCAGTAATCAGTCACAA-3'. The PCR assay was performed under the following conditions: initial denaturation step at 95 °C for 5 min, 34 cycles at 95 °C for 30 s, 30 s at 66 °C annealing temperature, 60 s extension at 72 °C and the final extension step for 5 min at 72 °C. The PCR amplification product of 343 bp, containing the polymorphic site, was digested with 1.5 U of *Bsm*AI restriction endonuclease (New England Biolabs, Ipswich, UK) in a final volume of 15 μL for 16 h at 37 °C. DNA fragments of sizes 236 and 107 bp indicate the G/G genotype, with 343, 236, and 107 being the G/T genotype and fragments of 343 bp the T/T genotype (Figure 2).

Digested products were evaluated on an 8% polyacrylamide gel. GeneRuler™ 100 bp (Fermentas, Hanover, MD, USA) or the ΦX174 DNA/BsuRI Marker (Fermentas, Hanover, MD, USA) was used as a molecular mass marker. Electrophoresis was carried out at 5 V/cm in TBE buffer and analysed under UV light following ethidium bromide staining. All PCR amplifications were conducted in a C1000 Thermal Cycler (Bio-Rad Laboratories, Hercules, CA, USA). Positive and negative (no template) controls were included in all sets. The accuracy of the genotyping was evaluated by performing duplicate analysis of 10% of samples, and the results were 100% concordant.

### 4.4. Statistical Analysis

To compare the distributions of demographic variables and selected risk factors between patients and controls, the chi-square (χ^2^) test was used. The Hardy–Weinberg equilibrium was checked using the χ^2^ test to compare the observed and expected genotype frequencies. The χ^2^ analysis was also used to test the significance of the differences between distributions of genotypes and alleles in KC/FECD patients and controls. The association between case-control status and each polymorphism, measured by the odds ratio (OR) and its corresponding 95% confidence interval (CI), was estimated using an unconditional multiple logistic regression model, both with and without adjustment for age, sex, co-occurrence of visual impairment, allergies, heart or vascular diseases and family status of KC/FECD. Statistical analysis was performed using the SigmaPlot software, version 11.0 (Systat Software, Inc., San Jose, CA, USA).

## 5. Conclusions

Our results suggest that the g.61564299G>T polymorphism of the *FEN1* gene may be associated with susceptibility to KC and FECD and be considered as markers in these diseases.
